# Systematic Analysis of the Association between Gut Flora and Obesity through High-Throughput Sequencing and Bioinformatics Approaches

**DOI:** 10.1155/2014/906168

**Published:** 2014-08-14

**Authors:** Chih-Min Chiu, Wei-Chih Huang, Shun-Long Weng, Han-Chi Tseng, Chao Liang, Wei-Chi Wang, Ting Yang, Tzu-Ling Yang, Chen-Tsung Weng, Tzu-Hao Chang, Hsien-Da Huang

**Affiliations:** ^1^Institute of Bioinformatics and Systems Biology, National Chiao Tung University, Hsinchu 300, Taiwan; ^2^Department of Obstetrics and Gynecology, Hsinchu Mackay Memorial Hospital, Hsinchu 300, Taiwan; ^3^Mackay Medicine, Nursing and Management College, Taipei 104, Taiwan; ^4^Department of Medicine, Mackay Medical College, New Taipei City 251, Taiwan; ^5^Tseng Han-Chi General Hospital, Nantou 542, Taiwan; ^6^Health GeneTech Corporation, Taoyuan 330, Taiwan; ^7^Graduate Institute of Biomedical Informatics, Taipei Medical University, Taipei 110, Taiwan; ^8^Department of Biological Science and Technology, National Chiao Tung University, Hsinchu 300, Taiwan; ^9^Center for Bioinformatics Research, National Chiao Tung University, Hsinchu 300, Taiwan; ^10^Department of Biomedical Science and Environmental Biology, Kaohsiung Medical University, Kaohsiung 807, Taiwan

## Abstract

Eighty-one stool samples from Taiwanese were collected for analysis of the association between the gut flora and obesity. The supervised analysis showed that the most, abundant genera of bacteria in normal samples (from people with a body mass index (BMI) ≤ 24) were* Bacteroides* (27.7%),* Prevotella* (19.4%),* Escherichia* (12%),* Phascolarctobacterium* (3.9%), and* Eubacterium* (3.5%). The most abundant genera of bacteria in case samples (with a BMI ≥ 27) were* Bacteroides* (29%),* Prevotella* (21%),* Escherichia* (7.4%),* Megamonas* (5.1%), and* Phascolarctobacterium* (3.8%). A principal coordinate analysis (PCoA) demonstrated that normal samples were clustered more compactly than case samples. An unsupervised analysis demonstrated that bacterial communities in the gut were clustered into two main groups: N-like and OB-like groups. Remarkably, most normal samples (78%) were clustered in the N-like group, and most case samples (81%) were clustered in the OB-like group (Fisher's *P*  value = 1.61E − 07). The results showed that bacterial communities in the gut were highly associated with obesity. This is the first study in Taiwan to investigate the association between human gut flora and obesity, and the results provide new insights into the correlation of bacteria with the rising trend in obesity.

## 1. Background

Enterobacteria, or gut microbiota, in the human gastrointestinal (GI) tract play important roles in the body's functions. For example, they can regulate immune responses and metabolic functions of the host [1–4]. The gut microbiota is frequently used to study the association between human health and an individual's lifestyle. In 2011, three major kinds of enterotypes consisting of* Bacteroides*,* Prevotella*, and* Ruminococcus* [5] provided new perspectives to classify individuals.* Ruminococcus* was reported to be an ambiguous enterotype, and the* Bacteroides* and* Prevotella* enterotypes were associated with dietary habits [6]. Animal protein and saturated fats were highly correlated with the* Bacteroides* enterotype. Low meat intake and plant-based nutrition with high carbohydrates were correlated with the* Prevotella* enterotype.

The Human Microbiome Project (HMP) [7] was launched by the US National Institutes of Health in 2008, and understanding the relationship between human health and microbiota that live in or on the human body was recognized as an important concept. To decipher this relationship, high-throughput sequencing, also called next-generation sequencing (NGS), supporting a large number of sequences, can be used to sequence 16S ribosomal (r)RNA to construct complex microbial community profiles. 16S rRNA is considered the standard for studying microbial communities and assigning taxonomy to bacteria. Compared to conventional polymerase chain reaction- (PCR-) based or culture-based methods, 16S rRNA sequencing by NGS can detect hundreds to thousands of bacteria at one time and offer relative quantification of the bacteria. Interactions between different bacterial communities and their environments can be comprehensively analyzed by metagenomics research. Associations between diseases and specific bacteria have been described in previous studies, for example, type 2 diabetes [8–11], irritable bowel syndrome (IBS) [12–14], and colorectal cancer (CRC) [15–17]. Moreover, some bacteria which are significantly associated with specific diseases were thought to be biomarkers for construction of a disease risk prediction model [8, 18].

Obesity is a major public health problem worldwide, and its prevalence is rapidly increasing [19]. Obesity is related to several disorders, including type 2 diabetes [20–23], cardiovascular disease [24–26], and cancer [26–28]. Recently, obesity was shown to be associated with an alteration of the gut microbiota, both in human [29–32] and animal models [30, 33, 34]. It was observed that a reduced proportion of the Bacteroidetes and increased proportion of the Firmicutes were associated with human obesity [30, 35, 36]. Also, an increase of* Actinobacteria* in obese individuals was reported [35]. In another study, amounts of Archaea and Methanobacteriales were positively correlated with obesity [37], and their amounts in obesity samples decreased or disappeared after gastric bypass surgery. In addition, another study also mentioned that the amounts of* Bifidobacterium* and* Ruminococcus* decreased in obesity samples [38]. However, some studies indicated that the ratio of the proportions of Bacteroidetes and Firmicutesis contradictory [39] or not associated [5, 40] with obesity.

With different ethnicities and regions, dietary habits and environmental factors can widely vary, and there is a lack of studies focusing on Taiwanese samples. Therefore, herein we collected 81 stool samples from Taiwanese for analysis of the association between gut flora and obesity. According to a study by Pan et al. [41], Taiwan adopted body mass index (BMI) values of 24 and 27 as the cutoff points for being overweight and obese, respectively. In this study, the stools of 36 obese (BMI ≥ 27) and 45 normal persons (BMI ≤ 24) were collected, and 16S rRNA sequencing was used to assess the association between obesity and the taxonomic composition of the gut microbiota.

## 2. Results

**Figure 1:**
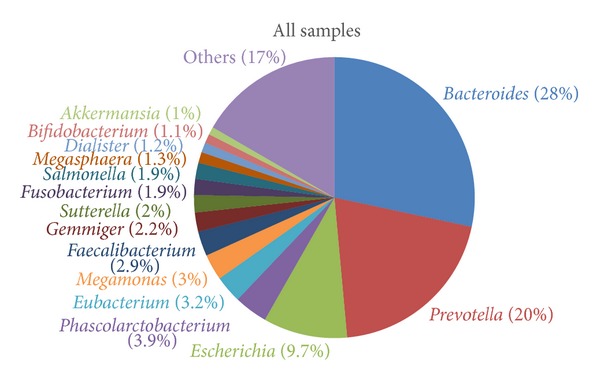
The distribution of genera among all samples.

Participant metadata are summarized in Table 1, and detailed sample profiles are given in Table S1 available online in Supplementary material at http://dx.doi.org/10.1155/2014/906168, including the number of reads, gender, age, height, weight, and BMI. In total, 4,152,740 sequence reads were obtained from the 81 samples, and a mean of 51,268 reads with a median read length of 125 bp was obtained per study participant. Sequence reads were processed through our taxonomic mapping process, and the distribution of genera in samples is depicted in Figure 1. The sequencing results showed that the most abundant genera in all samples were* Bacteroides* (28%),* Prevotella* (20%),* Escherichia* (9.7%),* Phascolarctobacterium* (3.9%),* Eubacterium* (3.2%),* Megamonas* (3%),* Faecalibacterium* (2.9%),* Gemmiger* (2.2%), and* Sutterella* (2%).

### 2.1. Unsupervised Clustering Analysis

**Figure 2:**
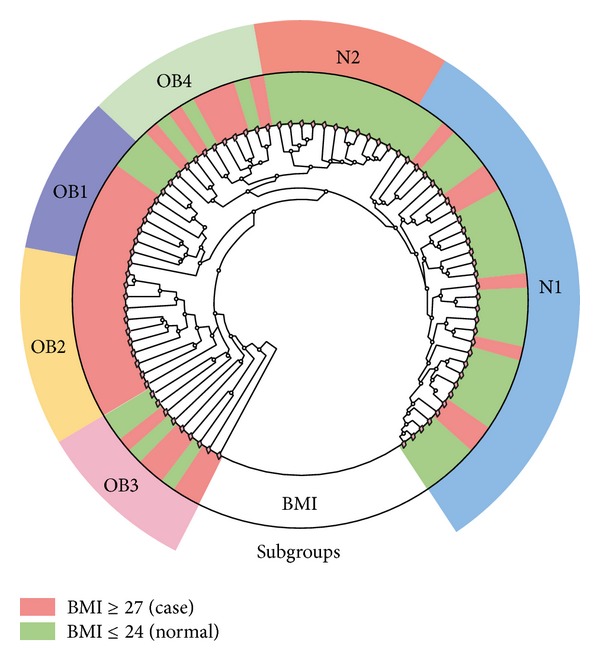
Bacterial communities in the samples.

**Figure 3:**
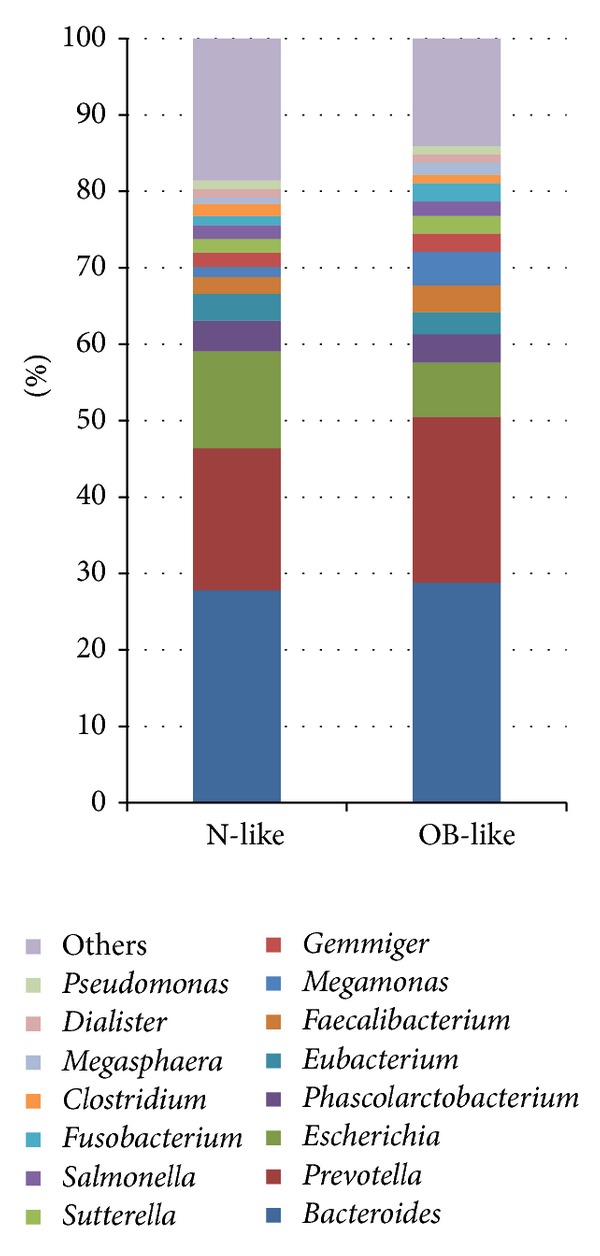
Relatively abundant genera in the N-like and OB-like groups.

**Figure 4:**
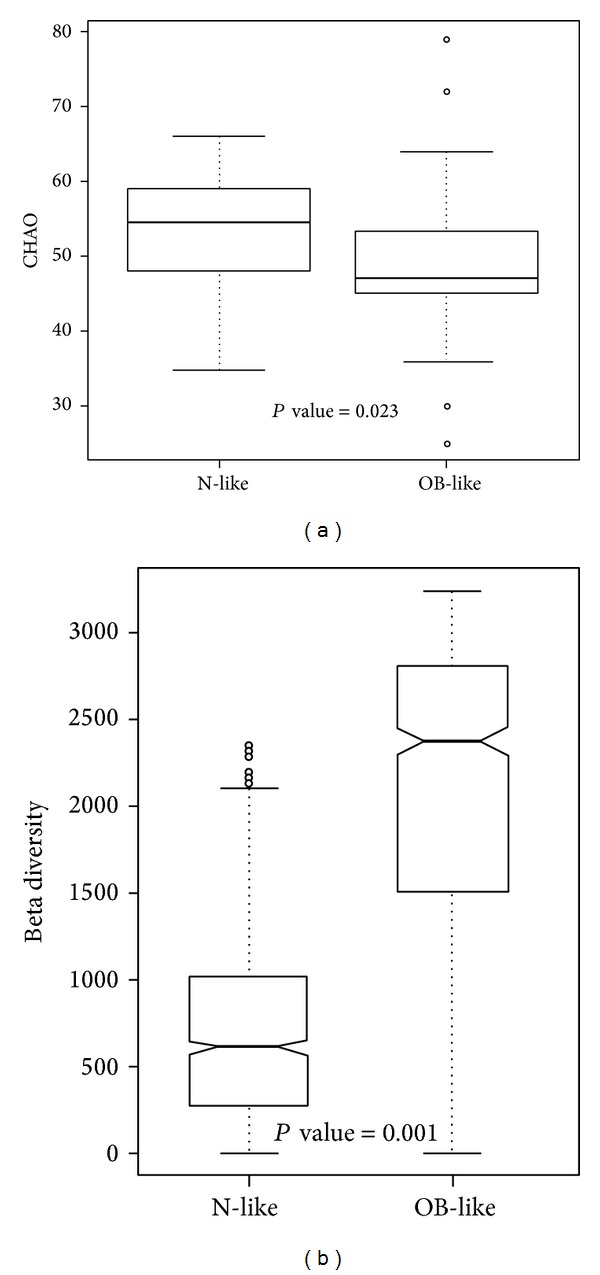
Unweighted (a) alpha diversity and (b) beta diversity of bacterial communities in the N-like and OB-like groups.

Hierarchical clustering was performed using the UniFrac unweighted distance, and gut bacterial communities and clinical values of each sample are shown in Figure 2. The results demonstrate that the bacterial communities in the gut were clustered into two main groups: an N-like group (including the N1 and N2 subgroups) and an OB-like group (including the OB1, OB2, OB3, and OB4 subgroups).  Figure 3 shows that the most abundant genera in N-like samples were* Bacteroides* (27.8%),* Prevotella* (18.6%),* Escherichia* (12.7%),* Phascolarctobacterium* (4%), and* Eubacterium* (3.5%). The most abundant genera in OB-like samples were* Bacteroides* (28.8%),* Prevotella* (21.7%),* Escherichia* (7.1%),* Megamonas* (4.4%), and* Phascolarctobacterium* (3.7%). Remarkably, most normal samples (78%) were clustered in the N-like group, and most case samples (81%) were clustered in the OB-like group (Fisher's *P* value = 1.61*E* − 07). The results showed that gut bacterial community types were highly associated with obesity. The genera diversity analysis showed that the bacterial communities in the N-like group exhibited significantly higher alpha diversity and lower beta diversity than those in the OB-like group (Figure 4).

### 2.2. Supervised Clustering Analysis

**Figure 5:**
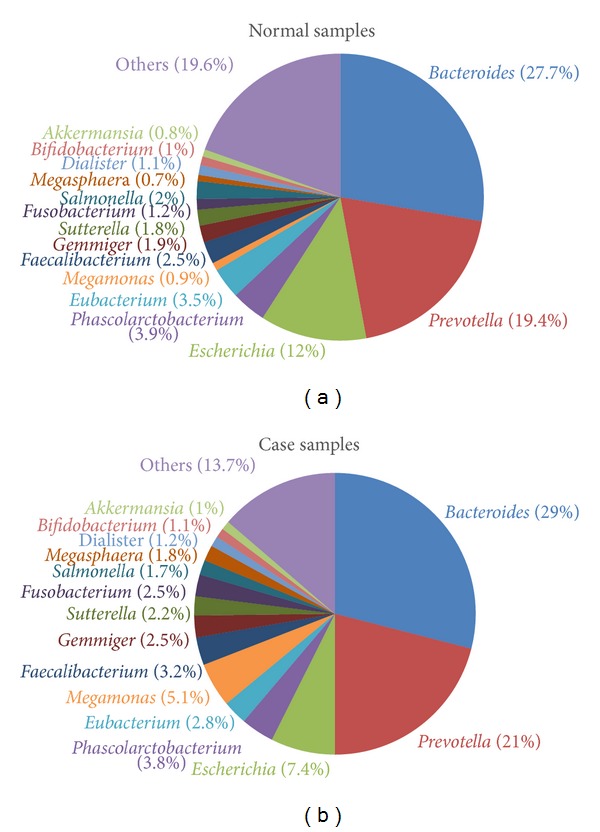
Relatively abundant genera in the normal and case samples.

To investigate the association between gut bacterial communities and obesity, 45 stool samples of participants with a BMI of ≤ 24 were defined as normal samples, and 36 samples of participants with a BMI ≥ 27 were used as case samples.  Figure 5 shows that the most abundant bacteria in normal samples were* Bacteroides* (27.7%),* Prevotella* (19.4%),* Escherichia* (12%),* Phascolarctobacterium* (3.9%), and* Eubacterium* (3.5%). The most abundant bacteria in case samples were* Bacteroides* (29%),* Prevotella* (21%),* Escherichia* (7.4%),* Megamonas* (5.1%), and* Phascolarctobacterium* (3.8%). Normal samples had a significantly higher proportion of* Escherichia*, while case samples had a higher proportion of* Megamonas*.

**Figure 6:**
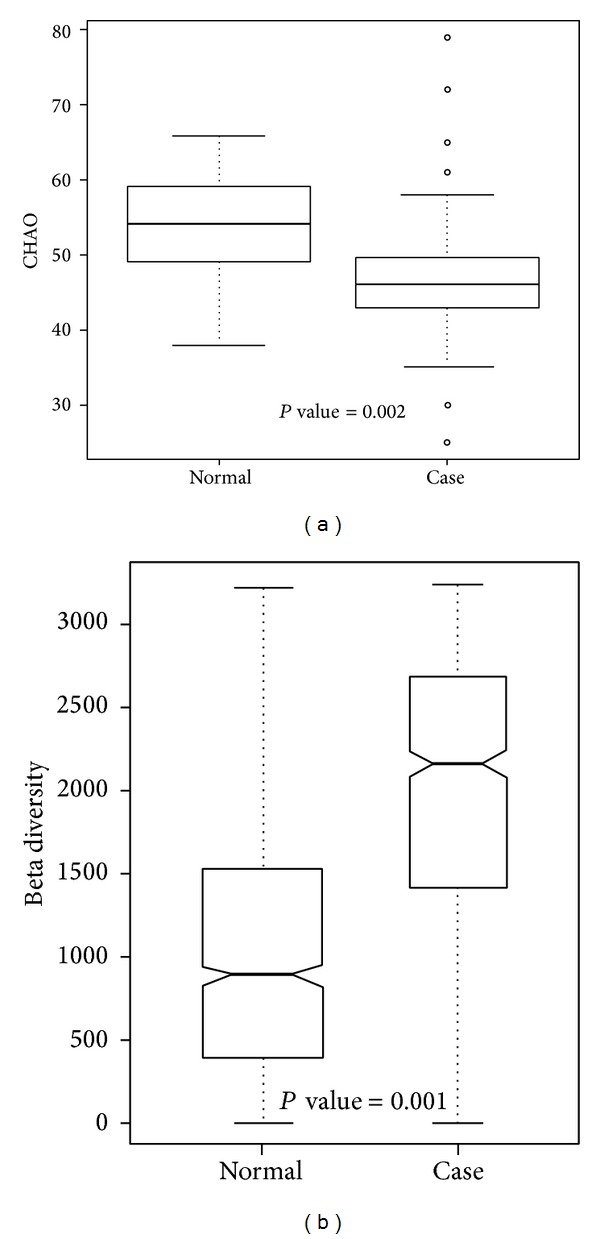
Unweighted (a) alpha diversity and (b) beta diversity of bacterial communities in case and control samples.

Genera with significantly different proportions between normal and case samples are listed in Table 2. Additionally, genera with a significantly different presence between normal and case samples are listed in Table 3, and significantly different species are also provided in Table S2. The genera of* Shewanella, Citrobacter, Cronobacter, Leclercia, Tatumella, and Acinetobacter* exhibited significant differences in both proportions and presence. Unweighted alpha and beta diversities of genera in the normal and case samples are shown in Figures 6(a) and 6(b), respectively. The results showed that the bacterial communities in normal samples exhibited significantly higher alpha diversity and lower beta diversity than those in case samples.

**Figure 7:**
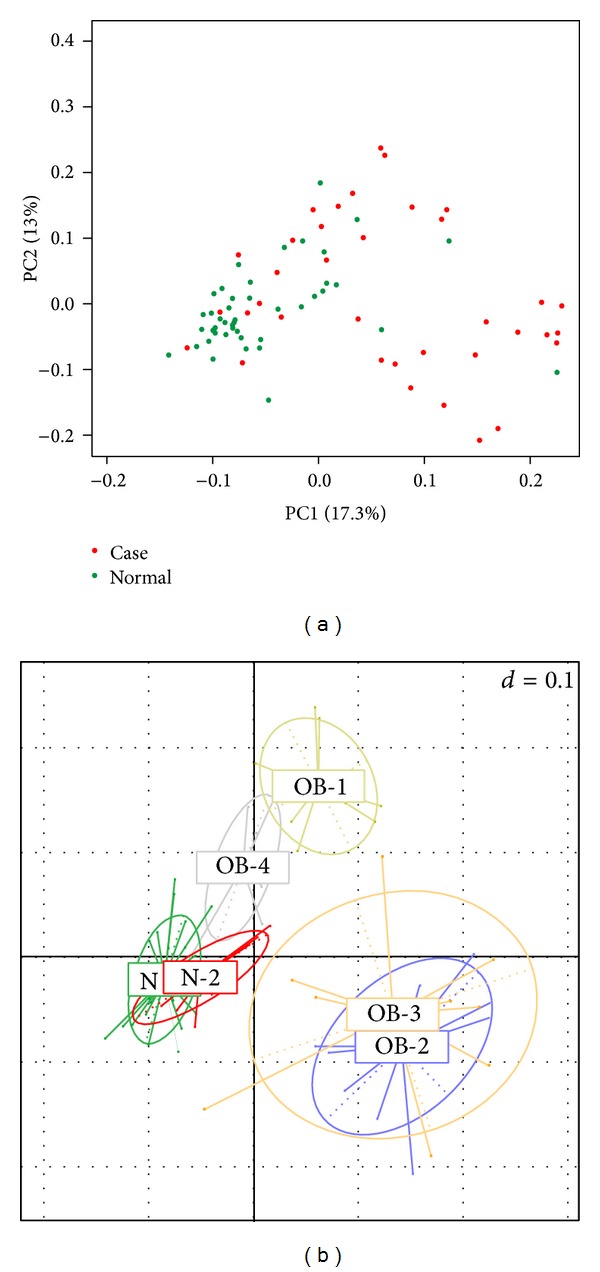
Unweighted principal coordinate analysis plot of (a) case and normal samples, (b) samples in N1, N2, OB1, OB2, OB3, and OB4 subgroups.

A PCoA of gut bacterial communities is shown in Figure 7. The results showed that most normal samples (green nodes) were located in the bottom left area, and case samples (red nodes) were spread in other areas (Figure 7(a)). Samples in the N1, N2, OB1, OB2, OB3, and OB4 subgroups are depicted in Figure 7(b). The results show that bacterial communities of N1 and N2 were highly associated with normal-weight individuals, and others were associated with obese individuals.

### 2.3. Potential Markers for Classification of Normal Weight and Obesity

**Figure 8:**
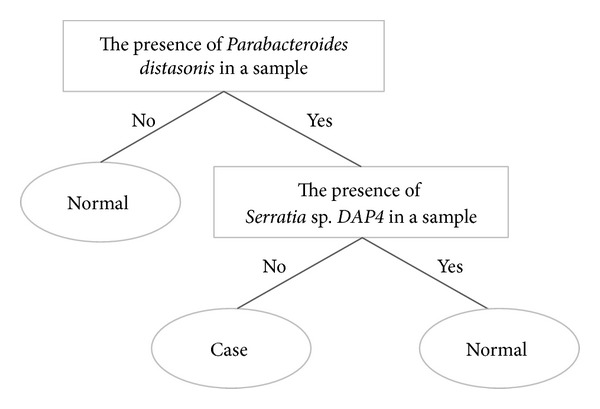
Classification rule and potential markers for discriminating between obese and normal-weight individuals.

The identified bacteria with statistical significance were used for rule-based clustering. Threefold cross-validation was used to evaluate the performance of the classification model. Two out of the significant species in Table S2,* Parabacteroides distasonis* and* Serratia *sp.* DAP4*, were selected as discriminating factors in the J48 decision tree. As shown in Figure 8, the classification rules are described as follows. (1) a sample is classified as normal if* Parabacteroides distasonis* was absent. (2) A sample with the presence of* Parabacteroides distasonis* and absence of* Serratia* sp.* DAP4* was classified as a case; otherwise, it was classified as normal. As shown in Table S3, the classifier performed well, and the area under the receiver operating characteristic curve (AUC) was 0.813. The results showed that* Parabacteroides distasonis* and* Serratia* sp.* DAP4* might be potential markers for further clinical analysis and investigation of obesity.

## 3. Discussion

Several relatively abundant genera were identified in samples (Figure 5), including* Bacteroides*,* Prevotella*,* Escherichia*,* Phascolarctobacterium*,* Eubacterium*,* Megamonas*,* Faecalibacterium*,* Gemmiger*,* Sutterella*,* Fusobacterium*,* Salmonella*,* Megasphaera*,* Dialister*,* Bifidobacterium*, and* Akkermansia*. In previous studies, the presence of* Bacteroides*,* Prevotella,* and* Sutterella* was negatively associated with obesity [36, 42, 43]. In other related gastrointestinal diseases,* Prevotella* was increased in children diagnosed with IBS [44].* Bacteroides*,* Eubacterium,* and* Prevotella* were increased, and* Faecalibacterium* was reduced in CRC patients [45, 46]. Increased* Bacteroides* and reduced* Eubacterium* and* Prevotella* were also found in a rat model of CRC [47].

At the genus level, the presence of* Acinetobacter*,* Aliivibrio*,* Marinomonas*,* Pseudoalteromonas*, and* Shewanella* had positive associations with obesity (Table 3).* Acinetobacter* is a genus of Gram-negative bacteria, and the species* Acinetobacter baumannii* is a key pathogen of infections in hospitals [48].* Aliivibrio* is a reclassified genus from the “*Vibrio fischeri* species group” [49], and species of* Aliivibrio* are symbiotic with marine animals or are described as fish pathogens [50–52].* Shewanella* is a genus of marine bacteria, and some species can cause infections [53, 54].* Lachnospira* [37],* Citrobacter* [43], and* Shigella *[43] were reported to be positively associated with obesity.* Lachnobacterium* [18] showed a negative association with obesity.

At the species level, the presence of* Parabacteroides distasonis*,* Lactobacillus kunkeei*,* Pseudoalteromonas piscicida*,* Shewanella algae*,* Marinomonas posidonica*, and* Aliivibrio fischeri* was positively associated with obesity (Table S2). Species of* Bacteroides* and* Parabacteroides* represent opportunistic pathogens in infectious diseases, and they are able to develop antimicrobial drug resistance [55].* Parabacteroides distasonis*, previously known as* Bacteroides distasonis* [56], is prominently found in the gut of healthy individuals [57]. It is also related to improved human bowel health release [58] and negatively associated with celiac disease [59]. Our results revealed a positive association between* Parabacteroides distasonis* and obesity.* Blautia producta* [60] and* Enterobacter cloacae* [61] were suggested to be related to a high-fat diet causing obesity in a mouse model.* Serratia* is a genus of Gram-negative, facultatively anaerobic, rod-shaped bacteria. In hospitals,* Serratia* species tend to colonize the respiratory and urinary tracts causing nosocomial infections [62, 63]. In related studies of the GI tract,* Serratia* increased in formula-fed mice [64] and was positively correlated with infants with colic [65].

In the alpha diversity analysis (Figure 6(a)), the Chao richness index between normal and case groups exhibited a significant difference (*P* = 0.002). This shows that bacterial communities in normal samples had a greater genera richness than those in case samples. Results of the beta diversity analysis (Figure 6(b)) showed that bacterial communities in normal samples were more similar than those in case samples. The unweighted PCoA plot (Figure 7) showed that bacterial communities in the N-like group (including N1 and N2) were highly associated with normal individuals, and bacterial communities in the OB-like group (including OB1, OB2, OB3, and OB4) were more associated with obese individuals. The unsupervised clustering heatmap of all samples (Figure S1(A)) was generated using Spearman correlations, and the results showed that most normal samples and most case samples were, respectively, clustered together, when all genera were used for clustering. However, when only relatively abundant genera were used for clustering, normal, and case samples were interwoven with each other (Figure S1(B)). This indicates that some genera found in small proportions might be important for distinguishing obese from normal individuals.

## 4. Conclusions

This is the first study in Taiwan to investigate the association between human gut microbiota and obesity using metagenomic sequencing. The results showed that bacterial communities in the gut were clustered into N-like and OB-like groups which were highly associated with normal and obese subjects, respectively. Several relatively abundant bacteria with significantly different distributions between normal and case samples were identified and used to establish a rule-based classification model. Although detailed functional roles or mechanisms of these bacteria are needed for further validation, the results provide new insights about bacterial communities in the gut with a rising trend of obesity.

## 5. Methods

### 5.1. Sample Collection and DNA Extraction

Eighty-one stool samples were collected by Sigma-transwab (Medical Wire) into a tube with Liquid Amies Transport Medium and stored at 4°C until being processed. Fresh faeces were obtained from participants, and DNA was directly extracted from stool samples using a QIAamp DNA Stool Mini Kit (Qiagen). A swab was vigorously vortexed and incubated at room temperature for 1 min. The sample was transferred to microcentrifuge tubes containing 560 *μ*L of Buffer ASL, then vortexed, and incubated at 37°C for 30 min. In addition, the suspension was incubated at 95°C for 15 min, vortexed, and centrifuged at 14,000 rpm for 1 min to obtain pelletized stool particles. Extraction was performed following the protocol of the QIAamp DNA Stool Mini Kit. DNA was eluted with 50 *μ*L Buffer AE, centrifuged at 14,000 rpm for 1 min, and then the DNA extract was stored at −20°C until being further analyzed.

### 5.2. Library Construction and Sequencing of the V4 Region of 16S rDNA

The PCR primers, F515 (5′-GTGCCAGCMGCCGCGGTAA-3′) and R806 (5′-GGACTACHVGGGTWTCTAAT-3′), were designed to amplify the V4 region of bacterial 16S rDNA as described previously [66]. Polymerase chain reaction (PCR) amplification was performed in a 50 *μ*L reaction volume containing 25 *μ*L 2x Taq Master Mix (Thermo Scientific), 0.2 *μ*M of each forward and reverse primer, and 20 ng of a DNA template. The reaction conditions included an initial temperature of 95°C for 5 min, followed by 30 cycles of 95°C for 30 s, 54°C for 1 min, and 72°C for 1 min, with a final extension of 72°C for 5 min. Next, amplified products were checked by 2% agarose gel electrophoresis and ethidium bromide staining. Amplicons were purified using the AMPure XP PCR Purification Kit (Agencourt) and quantified using the Qubit dsDNA HS Assay Kit (Qubit) on a Qubit 2.0 Fluorometer (Qubit), all according to the respective manufacturer's instructions. For V4 library preparation, Illumina adapters were attached to the amplicons using the Illumina TruSeq DNA Sample Preparation v2 Kit. Purified libraries were processed for cluster generation and sequencing using the MiSeq system.

### 5.3. Filtering 16S rRNA (rDNA) Sequencing Data for Quality

The FASTX-Toolkit (http://hannonlab.cshl.edu/fastx_toolkit) was used to process the raw fastq read data files from Illumina Miseq. The sequence quality criteria were as follows: (1) the minimum acceptable phred quality score of sequences was 30 with a score of >70% of sequence bases of ≥20; (2) after quality trimming from the sequence tail, sequences of >100 bp were retained, and they also had an acceptable phred quality score of 30; and (3) both forward and reverse sequencing reads which met the first and second requirements were retained for subsequent analysis. Sequencing reads from different samples were identified and separated according to specific barcodes in the 5′ end of the sequence (with two mismatches allowed).

### 5.4. Taxonomic Assignments of Bacterial 16S rRNA Sequences

Paired-end sequences were obtained, and their qualities were assessed using the FASTX-Toolkit. To generate taxonomic assignments, Bowtie2 was used to align sequencing reads against the collection of a 16S rRNA sequences database. A standard of 97% similarity against the database was applied. 16S rRNA sequences of bacteria were retrieved from the SILVA ribosomal RNA sequence database [67]. Following sequence data collection, sequences were extracted using V4 forward and reverse primers. To prevent repetitive sequence assignments, V4 sequences from SILVA were then clustered into several clusters by 97% similarity using UCLUST [68]. Results of the taxonomic assignment were filtered to retain assignments with >10 sequences.

### 5.5. Bacterial Community Analysis

After taxonomic assignment, an operational taxonomic unit (OTU) table was generated. To normalize the sample size of all samples, a rarefaction process was performed on the OTU table. Alpha and beta diversities were calculated based on a rarified OTU table. The Kolmogorov-Smirnov test and an analysis of variance (ANOVA) test with the Bonferroni correction were used to investigate significant differences between different sample groups. To observe relationships between samples and explore taxonomic associations, weighted and unweighted UniFrac [69] distance metrics were also generated based on the rarified OTU table. A principal coordinate analysis (PCoA) and unsupervised clustering were performed based on the UniFac distance matrix. To explore relationships between clinical features and different sample groups, Spearman's correlation coefficient and regression analysis were performed. The statistical analytical process was done in R language. The J48 machine learning method in Weka 3.6.7 [70] was used to construct a classification rule for discriminating between obese and normal individuals.

## Supplementary Material

Table S1: Characteristics of each sample
Table S2: Species with a significantly different presence between normal and case samples
S3: Performance of classification of obesity and normal using Parabacteroides distasonis and Serratia sp. DAP4
Figure S1: Unsupervised clustering heatmap using Speaman correlation with (A) all genera (B) relative abundant genera

## Figures and Tables

**Table 1:** Study participant characteristics and demographics.

*Participant characteristic *	
Gender (number of samples)	
Male	30
Female	51
Age (years)	
Range	20~89
Mean	41.2
Height (cm)	
Range	148.5~181
Mean	164
Weight (kg)	
Range	45~110
Mean	69.7
Body mass index (kg/m^2^) (number of samples)	
≤24	45
≥27	36

**Table 2:** Genera with significantly different proportions between normal and case samples.

Genus	Fold change (case/control)	KS test *P* value	ANOVA *P* value	Case mean	Control mean
*Collinsella *	0.56	0.01	0.37	0.001	0.002
*Barnesiella *	0.61	0.01	0.26	0.002	0.003
*Clostridium *	0.52	0.01	0.01	0.009	0.017
*Coprococcus *	0.54	0.04	0.37	0.001	0.001
*Lachnospira *	1.68	0.02	0.27	0.003	0.002
*Oscillibacter *	0.55	0.01	0.02	0.001	0.001
*Megamonas *	5.70	0.08	0.01	0.051	0.009
*Veillonella *	0.47	0.01	0.27	0.002	0.004
*Shewanella *	4.69	0.00	0.04	0.001	<0.001
*Citrobacter *	0.26	0.00	0.11	0.002	0.006
*Cronobacter *	0.08	0.00	0.00	<0.001	0.002
*Enterobacter *	0.38	0.00	0.07	0.001	0.002
*Erwinia *	0.18	0.00	0.00	<0.001	0.002
*Escherichia *	0.62	0.00	0.04	0.074	0.120
*Leclercia *	0.09	0.00	0.06	0.001	0.005
*Morganella *	0.05	0.01	0.07	<0.001	0.001
*Serratia *	0.02	0.00	0.00	<0.001	0.018
*Tatumella *	0.06	0.00	0.00	<0.001	0.001
*Halomonas *	3.51	0.00	0.08	0.005	0.002
*Acinetobacter *	116.08	0.00	0.03	0.002	<0.001

KS: Kolmogorov-Smirnov; ANOVA: analysis of variance.

**Table 3:** Genera with a significantly different presence between normal and case samples.

Genus	Presence Case/normal	Absence Case/normal	Fisher's test *P* value	Odds ratio (95% CI)
*Butyricimonas *	28/44	8/1	0.009	0.082 (0.002~0.664)
*Butyrivibrio *	0/11	36/34	0.001	0.088 (0.002~0.663)
*Lachnobacterium *	0/16	36/29	<0.001	0.052 (0.001~0.371)
*Lachnospira *	22/43	14/2	<0.001	0.076 (0.008~0.373)
*Syntrophococcus *	0/10	36/35	0.002	0.099 (0.002~0.764)
*Pectinatus *	0/14	36/31	<0.001	0.063 (0.001~0.460)
*Comamonas *	0/10	36/35	0.002	0.099 (0.002~0.764)
*Pseudoalteromonas *	12/1	24/44	<0.001	21.261 (2.837~956.295)
*Shewanella *	15/3	21/42	<0.001	9.703 (2.381~58.040)
*Citrobacter *	17/43	19/2	<0.001	0.043 (0.004~0.210)
*Cronobacter *	6/34	30/11	<0.001	0.068 (0.018~0.218)
*Leclercia *	8/36	28/9	<0.001	0.075 (0.021~0.233)
*Rahnella *	0/13	36/32	<0.001	0.070 (0.002~0.516)
*Shigella *	5/31	31/14	<0.001	0.076 (0.019~0.250)
*Tatumella *	2/23	34/22	<0.001	0.058 (0.006~0.273)
*Marinomonas *	12/1	24/44	<0.001	21.261 (2.837~956.295)
*Acinetobacter *	12/2	24/43	0.001	10.443 (2.070~103.809)
*Aliivibrio *	8/1	28/44	0.009	12.231 (1.505~568.466)

CI: confidence interval.

## References

[1] Goldsmith JR, Sartor RB (2014). The role of diet on intestinal microbiota metabolism: downstream impacts on host immune function and health, and therapeutic implications. *Journal of Gastroenterology*.

[2] Chen J, He X, Huang J (2014). Diet effects in gut microbiome and obesity. *Journal of Food Science*.

[3] Erejuwa OO, Sulaiman SA, Wahab MS (2014). Modulation of gut microbiota in the management of metabolic disorders: the prospects and challenges. *International Journal of Molecular Sciences*.

[4] Hold GL, Smith M, Grange C, Watt ER, El-Omar EM, Mukhopadhya I (2014). Role of the gut microbiota in inflammatory bowel disease pathogenesis: what have we learnt in the past 10 years?. *World Journal of Gastroenterology*.

[5] Arumugam M, Raes J, Pelletier E (2011). Enterotypes of the human gut microbiome. *Nature*.

[6] Wu GD, Chen J, Hoffmann C (2011). Linking long-term dietary patterns with gut microbial enterotypes. *Science*.

[7] Peterson J, Garges S, Giovanni M (2009). The NIH human microbiome project. *Genome Research*.

[8] Qin J, Li Y, Cai Z (2012). A metagenome-wide association study of gut microbiota in type 2 diabetes. *Nature*.

[9] Karlsson FH, Tremaroli V, Nookaew I (2013). Gut metagenome in European women with normal, impaired and diabetic glucose control. *Nature*.

[10] Zhang X, Shen D, Fang Z (2013). Human gut microbiota changes reveal the progression of glucose intolerance. *PLoS ONE*.

[11] Karlsson F, Tremaroli V, Nielsen J, Backhed F (2013). Assessing the human gut microbiota in metabolic diseases. *Diabetes*.

[12] Lyra A, Rinttilä T, Nikkilä J (2009). Diarrhoea-predominant irritable bowel syndrome distinguishable by 16S rRNA gene phylotype quantification. *World Journal of Gastroenterology*.

[13] Noor SO, Ridgway K, Scovell L (2010). Ulcerative colitis and irritable bowel patients exhibit distinct abnormalities of the gut microbiota. *BMC Gastroenterology*.

[14] Rajilić-Stojanović M, Biagi E, Heilig HGHJ (2011). Global and deep molecular analysis of microbiota signatures in fecal samples from patients with irritable bowel syndrome. *Gastroenterology*.

[15] Zhu Y, Michelle Luo T, Jobin C, Young HA (2011). Gut microbiota and probiotics in colon tumorigenesis. *Cancer Letters*.

[16] Hullar MA, Burnett-Hartman AN, Lampe JW (2014). Gut microbes, diet, and cancer. *Cancer Treatment and Research*.

[17] Hagland HR, Soreide K (2014). Cellular metabolism in colorectal carcinogenesis: influence of lifestyle, gut microbiome and metabolic pathways. *Cancer Letters*.

[18] Korpela K, Flint HJ, Johnstone AM (2014). Gut microbiota signatures predict host and microbiota responses to dietary interventions in obese individuals. *PLoS One*.

[19] Swinburn BA, Sacks G, Hall KD (2011). The global obesity pandemic: shaped by global drivers and local environments. *The Lancet*.

[20] Portero McLellan KC, Wyne K, Villagomez ET, Hsueh WA (2014). Therapeutic interventions to reduce the risk of progression from prediabetes to type 2 diabetes mellitus. *Therapeutics and Clinical Risk Management*.

[21] Everard A, Cani PD (2013). Diabetes, obesity and gut microbiota. *Best Practice and Research: Clinical Gastroenterology*.

[22] Bell JA, Kivimaki M, Hamer M (2014). Metabolically healthy obesity and risk of incident type 2 diabetes: a meta-analysis of prospective cohort studies. *Obesity Reviews*.

[23] Nagakubo D, Shirai M, Nakamura Y (2014). Prophylactic effects of the glucagon-like Peptide-1 analog liraglutide on hyperglycemia in a rat model of type 2 diabetes mellitus associated with chronic pancreatitis and obesity. *Comparative Medicine*.

[24] Kratz M, Baars T, Guyenet S (2013). The relationship between high-fat dairy consumption and obesity, cardiovascular, and metabolic disease. *European Journal of Nutrition*.

[25] Hinnouho GM, Czernichow S, Dugravot A (2014). Metabolically healthy obesity and the risk of cardiovascular disease and type 2 diabetes: The Whitehall II Cohort Study. *European Heart Journal*.

[26] Britton KA, Massaro JM, Murabito JM, Kreger BE, Hoffmann U, Fox CS (2013). Body fat distribution, incident cardiovascular disease, cancer, and all-cause mortality. *Journal of the American College of Cardiology*.

[27] Frezza EE, Wachtel MS, Chiriva-Internati M (2006). Influence of obesity on the risk of developing colon cancer. *Gut*.

[28] Nakamura K, Hongo A, Kodama J, Hiramatsu Y (2011). Fat accumulation in adipose tissues as a risk factor for the development of endometrial cancer. *Oncology Reports*.

[29] Remely M, Aumueller E, Jahn D, Hippe B, Brath H, Haslberger AG (2014). Microbiota and epigenetic regulation of inflammatory mediators in type 2 diabetes and obesity. *Beneficial Microbes*.

[30] Ley RE, Turnbaugh PJ, Klein S, Gordon JI (2006). Microbial ecology: human gut microbes associated with obesity. *Nature*.

[31] Shen J, Obin MS, Zhao L (2013). The gut microbiota, obesity and insulin resistance. *Molecular Aspects of Medicine*.

[32] Nieuwdorp M, Gilijamse PW, Pai N, Kaplan LM (2014). Role of the microbiome in energy regulation and metabolism. *Gastroenterology*.

[33] Turnbaugh PJ, Bäckhed F, Fulton L, Gordon JI (2008). Diet-induced obesity is linked to marked but reversible alterations in the mouse distal gut microbiome. *Cell Host and Microbe*.

[34] Bäckhed F, Ding H, Wang T (2004). The gut microbiota as an environmental factor that regulates fat storage. *Proceedings of the National Academy of Sciences of the United States of America*.

[35] Turnbaugh PJ, Hamady M, Yatsunenko T (2009). A core gut microbiome in obese and lean twins. *Nature*.

[36] Furet J, Kong L, Tap J (2010). Differential adaptation of human gut microbiota to bariatric surgery-induced weight loss: links with metabolic and low-grade inflammation markers. *Diabetes*.

[37] Zhang H, DiBaise JK, Zuccolo A (2009). Human gut microbiota in obesity and after gastric bypass. *Proceedings of the National Academy of Sciences of the United States of America*.

[38] Yin YN, Yu QF, Fu N, Liu XW, Lu FG (2010). Effects of four Bifidobacteria on obesity in high-fat diet induced rats. *World Journal of Gastroenterology*.

[39] Ley RE (2010). Obesity and the human microbiome. *Current Opinion in Gastroenterology*.

[40] The Human Microbiome Project Consortium (2012). Structure, function and diversity of the healthy human microbiome. *Nature*.

[41] Pan W, Flegal KM, Chang H, Yeh W, Yeh C, Lee W (2004). Body mass index and obesity-related metabolic disorders in Taiwanese and US whites and blacks: Implications for definitions of overweight and obesity for Asians. *The American Journal of Clinical Nutrition*.

[42] Santacruz A, Collado MC, García-Valdés L (2010). Gut microbiota composition is associated with body weight, weight gain and biochemical parameters in pregnant women. *British Journal of Nutrition*.

[43] Xiao S, Fei N, Pang X (2014). A gut microbiota-targeted dietary intervention for amelioration of chronic inflammation underlying metabolic syndrome. *FEMS Microbiology Ecology*.

[44] Rigsbee L, Agans R, Shankar V (2012). Quantitative profiling of gut microbiota of children with diarrhea-predominant irritable bowel syndrome. *The American Journal of Gastroenterology*.

[45] Wu N, Yang X, Zhang R (2013). Dysbiosis signature of fecal microbiota in colorectal cancer patients. *Microbial Ecology*.

[46] Chen W, Liu F, Ling Z, Tong X, Xiang C (2012). Human intestinal lumen and mucosa-associated microbiota in patients with colorectal cancer. *PLoS ONE*.

[47] Zhu Q, Jin Z, Wu W (2014). Analysis of the intestinal lumen microbiota in an animal model of colorectal cancer. *PLoS ONE*.

[48] Wang D, Yan D, Hou W, Zeng X, Qi Y, Chen J (2014). Characterization of bla_OxA-23_gene regions in isolates of *Acinetobacter baumannii*. *Journal of Microbiology, Immunology and Infection*.

[49] Urbanczyk H, Ast JC, Higgins MJ, Carson J, Dunlap PV (2007). Reclassification of *Vibrio fischeri*, *Vibrio logei*, *Vibrio salmonicida* and *Vibrio wodanis* as *Aliivibrio fischeri* gen. nov., comb. nov., *Aliivibrio logei* comb. nov., *Aliivibrio salmonicida* comb. nov. and *Aliivibrio wodanis* comb. nov. *International Journal of Systematic and Evolutionary Microbiology*.

[50] Ast JC, Urbanczyk H, Dunlap PV (2009). Multi-gene analysis reveals previously unrecognized phylogenetic diversity in *Aliivibrio*. *Systematic and Applied Microbiology*.

[51] Beaz-Hidalgo R, Doce A, Balboa S, Barja JL, Romalde JL (2010). *Aliivibrio finisterrensis* sp. nov., isolated from Manila clam, *Ruditapes philippinarum* and emended description of the genus Aliivibrio. *International Journal of Systematic and Evolutionary Microbiology*.

[52] Yoshizawa S, Karatani H, Wada M, Yokota A, Kogure K (2010). *Aliivibrio sifiae* sp. nov., luminous marine bacteria isolated from seawater. *Journal of General and Applied Microbiology*.

[53] Chen YS, Liu YC, Yen MY, Wang JH, Wann SR, Cheng DL (1997). Skin and soft-tissue manifestations of Shewanella putrefaciens infection. *Clinical Infectious Diseases*.

[54] Vignier N, Barreau M, Olive C (2013). Human infection with *Shewanella putrefaciens* and *S. algae*: report of 16 cases in Martinique and review of the literature. *American Journal of Tropical Medicine and Hygiene*.

[55] Boente RF, Ferreira LQ, Falcão LS (2010). Detection of resistance genes and susceptibility patterns in Bacteroides and Parabacteroides strains. *Anaerobe*.

[56] Sakamoto M, Benno Y (2006). Reclassification of Bacteroides distasonis, Bacteroides goldsteinii and Bacteroides merdae as Parabacteroides distasonis gen. nov., comb. nov., Parabacteroides goldsteinii comb. nov and Parabacteroides merdae comb. nov. *International Journal of Systematic and Evolutionary Microbiology*.

[57] Xu J, Mahowald MA, Ley RE (2007). Evolution of symbiotic bacteria in the distal human intestine.. *PLoS Biology*.

[58] Clarke JM, Topping DL, Christophersen CT (2011). Butyrate esterified to starch is released in the human gastrointestinal tract. *The American Journal of Clinical Nutrition*.

[59] Sánchez E, Donat E, Ribes-Koninckx C, Calabuig M, Sanz Y (2010). Intestinal *Bacteroides* species associated with coeliac disease. *Journal of Clinical Pathology*.

[60] Becker N, Kunath J, Loh G, Blaut M (2011). Human intestinal microbiota: characterization of a simplified and stable gnotobiotic rat model. *Gut Microbes*.

[61] Fei N, Zhao L (2013). An opportunistic pathogen isolated from the gut of an obese human causes obesity in germfree mice. *ISME Journal*.

[62] Hejazi A, Falkiner FR (1997). Serratia marcescens. *Journal of Medical Microbiology*.

[63] Patankar M, Sukumaran S, Chhibba A, Nayak U, Sequeira L (2012). Comparative in-vitro activity of cefoperazone-tazobactam and cefoperazone-sulbactam combinations against ESBL pathogens in respiratory and urinary infections. *Journal of Association of Physicians of India*.

[64] Carlisle EM, Poroyko V, Caplan MS, Alverdy J, Morowitz MJ, Liu D (2013). Murine gut microbiota and transcriptome are diet dependent. *Annals of Surgery*.

[65] de Weerth C, Fuentes S, Puylaert P, de vos WM (2013). Intestinal microbiota of infants with colic: development and specific signatures. *Pediatrics*.

[66] Caporaso JG, Lauber CL, Walters WA (2011). Global patterns of 16S rRNA diversity at a depth of millions of sequences per sample. *Proceedings of the National Academy of Sciences of the United States of America*.

[67] Pruesse E, Quast C, Knittel K (2007). SILVA: a comprehensive online resource for quality checked and aligned ribosomal RNA sequence data compatible with ARB. *Nucleic Acids Research*.

[68] Edgar RC (2010). Search and clustering orders of magnitude faster than BLAST. *Bioinformatics*.

[69] Lozupone C, Lladser ME, Knights D, Stombaugh J, Knight R (2011). UniFrac: an effective distance metric for microbial community comparison. *ISME Journal*.

[70] Hall M, Frank E, Holmes G, Pfahringer B, Reutemann P, Witten IH (2009). The WEKA data mining software: an update. *SIGKDD Explorations*.

