# Supplementary material for: Systematic Analysis of the Association between Gut Flora and Obesity through High-Throughput Sequencing and Bioinformatics Approaches
Source: Biomed Res Int. 2014 Aug 14;2014:906168. doi: 10.1155/2014/906168 (PMC4150407; doi:10.1155/2014/906168)
Supplement: Supplementary file 1 — Table S1: Characteristics of each sample Table S2: Species with a significantly different presence between normal and case samples S3: Performance of classification of obesity and normal using Parabacteroides distasonis and Serratia sp. DAP4 Figure S1: Unsupervised clustering heatmap using Speaman correlation with (A) all genera (B) relative abundant genera [file 906168.f1.pdf]

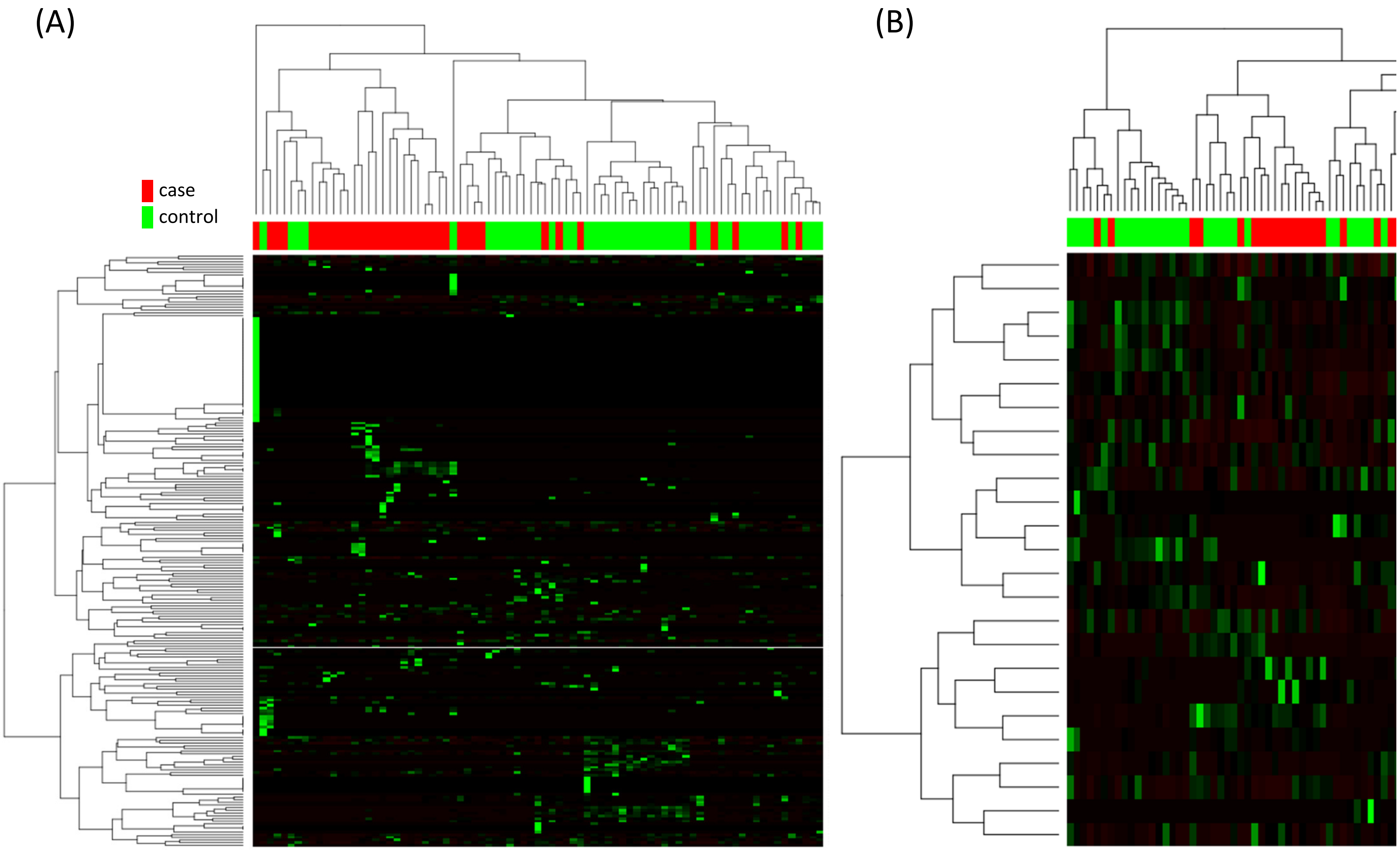

(A)
(B)
case
control

Table S3. Performance of classification of obesity and normal using *Parabacteroides distasonis* and *Serratia sp. DAP4*

	**Normal**	**Case**
**TP Rate**	0.889	0.722
**FP Rate**	0.278	0.111
**Precision**	0.800	0.839
**Recall**	0.889	0.722
**F-Measure**	0.842	0.776
**AUC**	0.813	0.813

TP: true positive, FP: false positive, Precision: TP/(TP+FP), Recall=TP/(TP+FN), FN: false negative, F-Measure= 2*Precision*Recall/(Precision+Recall), AUC: area under the ROC curve, ROC: receiver operating characteristic

Table S2. Species with a significantly different presence between normal and case samples

Genus	Presence case/normal	Absence case/normal	Fisher's test p-value	Odds ratio (95% CI)
**Butyricimonas virosa**	28/44	8/1	0.009	0.082 (0.002-0.664)
**Parabacteroides distasonis**	27/5	9/40	<0.001	22.671 (6.443-97.631)
**Parabacteroides merdae**	6/30	30/15	<0.001	0.103 (0.029-0.322)
**Alistipes shahii**	22/43	14/2	<0.001	0.076 (0.008-0.373)
**Lactobacillus kunkeei**	7/0	29/45	0.002	10.583 (1.256-498.823)
**Clostridium aldenense**	6/34	30/11	<0.001	0.068 (0.018-0.218)
**Clostridium sp. Clone-17**	0/15	36/30	<0.001	0.057 (0.001-0.412)
**Eubacterium desmolans**	2/17	34/28	<0.001	0.099 (0.010-0.475)
**Blautia product**	2/20	34/25	<0.001	0.076 (0.008-0.358)
**Lachnobacterium bovis**	0/16	36/29	<0.001	0.052 (0.001-0.371)
**uncultured Lachnospira sp.**	22/43	14/2	<0.001	0.076 (0.008-0.373)
**Syntrophococcus sucromutans**	0/10	36/35	0.002	0.099 (0.002-0.764)
**Ruminococcus albus**	0/11	36/34	<0.001	0.088 (0.002-0.663)
**Ruminococcus sp. ID1**	2/20	34/25	<0.001	0.076 (0.008-0.358)
**Pectinatus cerevisiiphilus**	0/14	36/31	<0.001	0.063 (0.001-0.460)
**Comamonas testosteroni**	0/10	36/35	0.002	0.099 (0.002-0.764)
**Sutterella stercoricanis**	28/44	8/1	0.009	0.082 (0.002-0.664)
**Sutterella wadsworthensis**	27/45	9/0	<0.001	0.069 (0.001-0.542)
**Desulfovibrio sp. enrichment culture clone Jdgsrb011**	1/11	35/34	0.010	0.090 (0.002-0.683)
**Pseudoalteromonas piscicida**	12/1	24/44	<0.001	21.261 (2.837-956.295)
**Shewanella algae**	15/3	21/42	<0.001	9.703 (2.381-58.040)

**Citrobacter amalonaticus**	14/41	22/4	<0.001	0.065 (0.014-0.234)
**Citrobacter sp. I91-10**	0/10	36/35	0.002	0.099 (0.002-0.764)
**Cronobacter sakazakii**	6/34	30/11	<0.001	0.068 (0.018-0.218)
**Enterobacter cloacae**	3/22	33/23	<0.001	0.098 (0.017-0.381)
**Enterobacter hormaechei**	0/16	36/29	<0.001	0.052 (0.001-0.371)
**Erwinia rhapontici**	2/16	34/29	0.001	0.109 (0.011-0.526)
**Leclercia adecarboxylata**	8/36	28/9	<0.001	0.075 (0.021-0.233)
**Rahnella sp. EMA-83**	0/13	36/32	<0.001	0.070 (0.002-0.516)
**Serratia proteamaculans**	0/17	36/28	<0.001	0.047 (0.001-0.336)
**Serratia sp. DAP4**	0/16	36/29	<0.001	0.052 (0.001-0.371)
**Serratia sp. M1**	0/16	36/29	<0.001	0.052 (0.001-0.371)
**uncultured Serratia sp.**	0/17	36/28	<0.001	0.047 (0.001-0.336)
**uncultured Shigella sp.**	5/31	31/14	<0.001	0.076 (0.019-0.250)
**Tatumella sp. L3-179**	0/16	36/29	<0.001	0.052 (0.001-0.371)
**Marinomonas posidonica**	12/1	24/44	<0.001	21.261 (2.837-956.295)
**Aliivibrio fischeri**	8/1	28/44	0.009	12.231 (1.505-568.466)

Table S1. Characteristics of each sample

	Reads#	Gender	Age	Height (cm)	Weight (kg)	BMI
S1	61780	M	50	169	67	23.5
S2	29956	F	26	158	53	21.2
S3	14271	M	68	168	60	21.3
S4	20330	M	24	173	64	21.4
S5	18597	F	30	165	62	22.8
S6	17055	F	26	160	50	19.5
S7	135847	F	43	164	62	23.1
S8	22941	M	54	172	54	18.3
S9	18387	F	27	167	56	20.1
S10	63130	M	36	173	68	22.7
S11	72530	F	30	159	59	23.3
S12	34060	F	26	163	53	19.9
S13	43321	F	28	164	55	20.4
S14	51859	F	27	162	46	17.5
S15	54715	M	24	171	70	23.9
S16	58688	M	27	175	68	22.2
S17	31877	M	23	174	70	23.1
S18	89940	M	52	165	65	23.9
S19	69643	F	24	161	50	19.3
S20	48747	M	57	165	65	23.9
S21	61752	M	34	176	74	23.9
S22	65812	F	40	169	63	22.1
S23	23770	M	32	169	50	17.5
S24	64988	M	27	169.5	62	21.6
S25	55832	F	54	155	49	20.4
S26	13758	F	33	155	46	19.1
S27	65042	F	35	160	60	23.4
S28	31523	F	58	163	56	21.1
S29	115092	M	35	178	51	16.1
S30	84928	M	67	168	67	23.7
S31	51986	F	54	155	47.5	19.8
S32	102450	F	67	155	56	23.3
S33	114501	F	43	163	53	19.9
S34	42232	F	54	161	62	23.9
S35	18351	F	34	153	51	21.8
S36	47313	F	35	168.5	47	16.6
S37	49205	M	63	169.5	68	23.7
S38	84415	M	65	165	54	19.8
S39	34592	F	41	161	51	19.7
S40	31054	F	49	157	45	18.3
S41	37027	F	60	157	52.5	21.3

S42	24673	F	52	163	59	22.2
S43	15504	M	50	170	67	23.2
S44	58194	F	42	158	52	20.8
S45	71320	M	26	168	61	21.6
S46	87415	F	63	160	70	27.3
S47	21886	M	20	161	70	27
S48	16478	M	42	174	86	28.4
S49	16830	F	59	158	70	28
S50	16528	F	27	166	86	31.2
S51	32115	M	78	163	79	29.7
S52	14065	M	33	176	98	31.6
S53	52206	M	62	163	74	27.9
S54	29772	M	43	170	81	28
S55	94893	M	23	171	87	29.8
S56	55794	M	49	175	95	31
S57	57077	F	35	160	80	31.3
S58	56127	F	26	153	72.5	31
S59	52021	F	46	159	99	39.2
S60	54294	F	41	159	100.4	39.7
S61	35979	F	47	164	95	35.3
S62	45421	F	22	160	90.4	35.3
S63	51395	F	29	158	103	41.3
S64	25697	F	37	154	79.9	33.7
S65	97069	M	34	170	97	33.6
S66	61917	F	26	166	82.9	30.1
S67	62806	F	20	160	84.5	33
S68	44477	F	41	159	74	29.3
S69	52976	F	35	170	110	38.1
S70	53911	F	55	152	78	33.8
S71	35613	F	48	165	85.1	31.3
S72	45512	F	37	158	70.3	28.2
S73	61104	M	48	165	85	31.2
S74	48071	F	42	148.5	69.2	31.4
S75	68550	F	33	151	68	29.8
S76	65130	F	48	159	88	34.8
S77	85723	M	33	175	95	31
S78	53911	F	89	181	89	27.2
S79	53743	F	43	166	88.7	32.2
S80	48547	F	33	158	71.7	28.7
S81	74699	F	42	158	90.4	36.2
